# HIV drug nucleoside reverse transcriptase inhibitors as promising anti-inflammation therapeutics by targeting P2X7-dependent large pore formation: one stone for two birds?

**DOI:** 10.3389/fphar.2015.00038

**Published:** 2015-02-25

**Authors:** Lin-Hua Jiang

**Affiliations:** Faculty of Biological Sciences, School of Biomedical Sciences, University of LeedsLeeds, UK

**Keywords:** nucleoside reverse transcriptase inhibitors, anti-inflammation, P2X7 receptor, large pore formation, HIV drugs

Nucleoside reverse transcriptase inhibitors (NRTIs) have been the backbones of HIV therapy since introduced in the late 1980s. These HIV drugs act by blocking the viral reverse transcriptase activity that is crucial in the HIV life cycle. In a recent study published by Science, Fowler et al. (2014) have shown that such antiretroviral agents can inhibit P2X7 receptor (P2X7R)-mediated *Alu* RNA-induced activation of caspase-1 in retinal pigment epithelial (RPE) cells and ATP-induced activation of caspase-1 in macrophage cells, independently of reverse transcriptase inhibition.

P2X7R belongs to the ATP-gated ion channel P2XR family and is highly expressed in immune and epithelial cells (North, 2002). P2X7R activation requires submillimolar ATP and the P2X7R is thus well placed as a molecular sensor detecting extracellular ATP, an endogenous danger associated with tissue damage and inflammation. It is firmly established that P2X7R in macrophage cells plays an essential role in mediating ATP-induced assembly of the multi-protein complex NLRP3 inflammasome, activation of caspase-1 and generation of interleukin (IL)-1β, a key proinflammatory cytokine in innate immunity. Such signaling mechanisms, if not resolved in time, can lead to numerous inflammatory conditions. P2X7R antagonists have been passionately exploited as anti-inflammation therapeutics. Several drug discovery programs have discovered a number of structurally novel compounds as P2X7R antagonists with drug-like properties. Such compounds show promising anti-inflammation efficacy in preclinical studies using animal models, but recent clinical trials treating rheumatoid arthritis have been rather disappointing. Non-coding *Alu*-derived RNAs are accumulated in the retinal pigment epithelium, and *Alu* RNAs-induced RPE cell death underlies geographic atrophy and choroidal neovascularization, two distinctive forms of aged-related macular degeneration responsible for loss of vision in the elderly people. The same group shows in another study that *Alu* RNAs can stimulate the P2X7R activity via undefined mechanisms to activate the NLRP3 inflammasome and caspase-1. In this recent study, they provide evidence to show that NRTIs inhibit P2X7R-mediated activation of caspase-1 in RPE cells and in LPS-primed macrophage cells induced by *Alu* RNAs and ATP, respectively. Furthermore, they have demonstrated that such HIV drugs exhibit significant anti-inflammation efficacy in mouse models of geographic atrophy, choroidal neovascularization, graft-vs-host diseases, and sterile liver inflammation. This is a truly exciting finding as many clinically proved HIV drugs can be readily tested as anti-inflammation therapeutics.

P2X7R, like all other P2XRs, functions as a channel that opens upon brief stimulation to permeate small physiological cations (North, 2002). However, it is known from the conception of the P2XR research that P2X7R is functionally exceptional; prolonged activation can induce a large pore that passes molecules in size of up to 900 Da and can eventually lead to cell death, thus formerly named the cytolytic P2Z receptor (Surprenant et al., 1996). Nearly 20 years on from identification as the last member of the P2XR family, the molecular mechanisms underpinning such striking plasticity in permeability is still puzzling. A recent study unequivocally demonstrates that the P2X7R small cationic channel can dilate to become the large pore (Browne et al., 2013). There is compelling evidence to support that P2X7R-dependent formation of the intrinsic large pore in macrophage cells engages pannexin-1, and particularly requires an interaction of pannexin-1 with the unique C-terminus of the P2X7R (Pelegrin and Surprenant, 2006; Sorge et al., 2012). The genes encoding the mammalian P2X7Rs and particularly human P2X7R are prolific with non-synonymous single nucleotide polymorphisms (NS-SNP), and NS-SNP mutations can significantly alter the small cationic channel, the large pore, or both functionalities. For example, the NS-SNP P451L mutation in the C-terminus of the mouse P2X7R totally abolishes the large pore formation without altering the small cationic channel function in macrophage cells (Sorge et al., 2012). Furthermore, the mice expressing P2X7R containing Leu^451^ are considerably less susceptible to inflammatory pain compared to the mice expressing the P2X7R harboring Pro^451^, explicitly indicating that the large pore but not the small cationic channel is crucial in dictating the severity of inflammatory disease. Such notion is echoed by the intriguing mechanisms of inhibition of the HIV drugs revealed in the study by Fowler et al. (2014). P2X7R activation is required for both *Alu* RNAs-induced caspase-1 activation in RPE cells and ATP-induced caspase-1 activation in LPS-primed macrophage cells. Blockage of the P2X7R would be the simplest answer, but the truth seems never so straightforward. In HEK293 cells heterologously expressing rat or mouse P2X7R, A438079, a P2X7R antagonist, completely prevented the small cationic channel and large pore. In striking contrast, the HIV drug tested reduced partially but significantly the large pore formation without effect on the small cationic channel, supporting preferred targeting of the large pore (Fowler et al., 2014). This is consistent with no effect on *Alu* RNAs-induced caspase-1 activation in RPE cells of calmidazolium, a P2X7R inhibitor known to preferentially block the open small cationic channel but not the large pore or cell death. Surprisingly, *Alu* RNAs-induced caspase-1 activation remained unaltered in RPE cells from the pannexin-1 deficient mice, suggesting that pannexin-1 is not required as reported previously for ATP-induced caspase-1 activation in LPS-primed macrophage cells (Qu et al., 2011). The intriguing twist was that *Alu* RNAs-induced capase-1 activation was abolished by ^10^Pan, a mimetic peptide thought to block pannexin-1 dependent large pore formation in macrophage cells (Pelegrin and Surprenant, 2006; Sorge et al., 2012). Such remarkable inhibition was however attributed to be a non-specific effect on formation of a large pore independent of pannexin-1 (Fowler et al., 2014). This interpretation requires more vigorous examination, for example, using RPE cells from the mice expressing the P2X7R with Pro^451^ or Leu^451^ and using the Pro^451^-containing competing peptide to disrupt the interaction between pannexin-1 and P2X7R, as shown in the aforementioned study (Sorge et al., 2012). Regardless, these recent studies, examining HIV drug inhibition of P2X7R-mediated caspase-1 activation (Fowler et al., 2014) and P2X7R-mediated inflammatory pain (Sorge et al., 2012), provide consistent evidence to suggest that the large pore functionality is more crucial than the small cationic channel in P2X7R-mediated inflammation. Such a disease mechanistic insight is fundamentally important that needs to be borne in mind for development and certainly clinical trials of anti-inflammation therapeutics targeting the P2X7R.

## Conflict of interest statement

The author declares that the research was conducted in the absence of any commercial or financial relationships that could be construed as a potential conflict of interest.
